# Fingolimod for Irradiation-Induced Neurodegeneration

**DOI:** 10.3389/fnins.2019.00699

**Published:** 2019-07-09

**Authors:** Judith Metzdorf, Zaynab Hobloss, Sibylle Schlevogt, Ilya Ayzenberg, Sarah Stahlke, Xiomara Pedreiturria, Steffen Haupeltshofer, Ralf Gold, Lars Tönges, Ingo Kleiter

**Affiliations:** ^1^Department of Neurology, St. Josef-Hospital, Ruhr-University, Bochum, Germany; ^2^Department of Neurology, I.M. Sechenov First Moscow State Medical University, Moscow, Russia; ^3^Marianne-Strauß-Klinik, Behandlungszentrum Kempfenhausen für Multiple Sklerose Kranke, Berg, Germany

**Keywords:** fingolimod, neuroprotection, X-ray, radiation, neurogenesis, neuronal precursor cells

## Abstract

**Background:**

Cranial irradiation is a common therapy for the treatment of brain tumors, but unfortunately patients suffer from side effects, particularly cognitive impairment, caused by neurodegenerative and neuroinflammatory mechanisms. Finding a therapeutic agent protecting hippocampal neurons would be beneficial. Fingolimod (FTY720), a sphingosine-1-phosphate receptor modulator approved for multiple sclerosis, is an immunosuppressant and known to enhance proliferation and differentiation of neuronal precursor cells (NPCs).

**Objectives:**

To investigate whether pre-treatment with FTY720 protects NPCs *in vitro* and *in vivo* from irradiation-induced damage.

**Methods:**

Neuronal precursor cells were isolated from E13 C57BL/6 wildtype mice, treated at day 0 of differentiation with FTY720 and irradiated on day 6 with 1 Gy. NPCs were analyzed for markers of cell death (PI, caspase-3), proliferation (Ki67), and differentiation (DCX, βIII-tubulin). Adult C57BL/6 wildtype mice were treated with FTY720 (1 mg/kg) and received a single dose of 6 Gy cranial irradiation at day 7. Using immunohistochemistry, we analyzed DCX and BrdU as markers of neurogenesis and Iba1, GFAP, and CD3 to visualize inflammation in the dentate gyrus (DG) and the subventricular zone (SVZ). B6(Cg)-Tyrc-2J/J DCX-luc reporter mice were used for bioluminescence imaging to evaluate the effect of FTY720 on neurogenesis in the DG and the spinal cord of naïve mice.

**Results:**

FTY720 protected NPCs against irradiation induced cell death *in vitro*. Treatment with FTY720 dose-dependently reduced the number of PI^+^ cells 24 and 96 h after irradiation without effecting proliferation or neuronal differentiation. *In vivo* treatment resulted in a significant survival of DCX^+^ neurons in the DG and the SVZ 4 weeks after irradiation as well as a slight increase of proliferating cells. FTY720 inhibited microglia activation 24 h after X-ray exposure in the DG, while astrocyte activation was unaffected and no lymphocyte infiltrations were found. In naïve mice, FTY720 treatment for 4 weeks had no effect on neurogenesis.

**Conclusion:**

FTY720 treatment of NPCs prior to X-ray exposure and of mice prior to cranial irradiation is neuroprotective. No effects on neurogenesis were found.

## Introduction

Cranial irradiation is a common therapy for primary and metastatic brain tumors, however, patients often suffer from side effects like cognitive impairments and depression due to late delayed brain injury (Son et al., 2015b). Damage to neurons in the DG, one of the neurogenic niches in the adult brain, is an important mechanism of radiation-induced CNS symptoms (Rola et al., 2004). Therapeutic strategies to either directly or indirectly protect neurons against radiation-induced damage or to promote neurogenesis would be desirable. Antioxidative effects and inhibition of apoptosis can directly affect neuronal survival, while reduction of deleterious neuroinflammation is an example of an indirect mechanism of neuroprotection.

Previous experimental studies demonstrated that environmental enrichment and voluntary running can induce neurogenesis and lead to recovery of cognitive function after irradiation (Fan et al., 2007; Wong-Goodrich et al., 2010). The preventive use of neuroprotective substances is an alternative approach to ameliorate radiation-induced injury. It has been shown that treatment with valproic acid (VPA) or lithium prior to irradiation protects hippocampal neurons from cell death and attenuates cognitive decline in preclinical models (Khasraw et al., 2012; Thotala et al., 2015). Another potentially therapeutic agent is the sphingosine-1-phosphate receptor modulator fingolimod (FTY720). Fingolimod, approved in 2011, was the first oral therapeutic agent for relapsing remitting multiple sclerosis (Chun and Brinkmann, 2011). After phosphorylation *in vivo*, FTY720P is able to target four of the five sphingosine-1-phosphate receptors (S1PR1, S1PR3, S1PR4, S1PR5). FTY720 is predominantly known for its immunosuppressive function by preventing the egress of lymphocytes from lymph nodes, however, S1PRs are widely expressed on all cells of the CNS, except S1PR4 (Brinkmann et al., 2002). Due to its lipophilic nature, FTY720 is able to cross the blood-brain-barrier. Previous experimental studies showed positive effects of FTY720 on embryonic and adult neurogenesis. Treatment with FTY720 increases proliferation and migration of embryonic neural stem cells *in vitro* as well as differentiation toward protoplasmic astrocytes (Tan et al., 2016). *In vivo* FTY720 enhances the production of neuronal precursor cells (NPCs) in the hippocampus of healthy mice accompanied by improved memory and learning abilities (Efstathopoulos et al., 2015; Sun et al., 2016). In a model of excitotoxic cell death, pretreatment with FTY720 increased neuronal viability mediated by S1PR1 (Di Menna et al., 2013).

We hypothesized that treatment with FTY720 prior to cranial irradiation has neuroprotective effects. We investigated whether FTY720 influences cell death, proliferation and differentiation of embryonic neuronal precursor cells (NPCs) *in vitro* and neurogenesis and inflammation in the neurogenic niches of the irradiated adult rodent brain.

## Materials and Methods

### Mice and Ethics Statement

C57BL/6 wildtype and B6(Cg)-Tyrc-2J/J DCX-luc reporter mice (Couillard-Despres et al., 2008) were kept at the central animal facility of the Center for Clinical Research [Zentrum für Klinische Forschung I (ZKFI), Zentrale Tierversuchsanstalt der Medizin (ZVM)] at the Ruhr-Universität Bochum under a 12 h night and day cycle with food and water *ad libitum*. All experiments were approved by the State Agency for Nature, Environment and Consumer Protection of North Rhine-Westphalia (LANUV: 84-02.04.2013.A038; 84-02.04.2015.A478) and performed in compliance with all applicable guidelines.

### Generation, Treatment, and Irradiation of Mouse Embryonic Precursor Cells

Embryonic NPCs from E13 C57BL/6 wildtype mice were cultured as neurospheres in a mixture of DMEM- and F12-medium (1:1, Sigma-Aldrich, St. Louis, MO, United States) containing 2% B27 (Life Technologies, Carlsbad, CA, United States), 1% PenStrep (Life Technologies), 20 ng/ml EGF and 20 ng/ml FGF (PAN-Biotech, Aidenbach, Germany). For experiments involving immunocytochemistry, NPCs were cultured as monolayers on coverslips in a 24-well-plate with a density of 30,000 cells per well. Coverslips were first coated with poly-D-lysin (10 μg/ml, Sigma-Aldrich) over night and then incubated with laminin (10 μg/ml, Sigma-Aldrich) for 2 h at 37°C. For qRT-PCR and FACS analysis 400,000 single cells were plated directly on coated 6-well-plates. To induce differentiation, 1% (FBS, Thermo Fisher Scientific, Waltham, MA, United States) was added to the culture medium, without EGF and FGF. Directly after seeding, phosphorylated FTY720 (Cayman Chemical, MI, United States) was added at different concentrations (1, 10, and 100 nM) to the cultures. After 6 days of differentiation the cells received a medium change and fresh FTY720P at the same concentration. On the same day, the cultures were exposed to a single dose of 1 Gy (220 kv and 7 mA) radiation with an X-ray machine RT250 (CHF Müller, Hamburg, Germany) at the RUBION center of the Ruhr-University Bochum. Untreated control groups, sham-irradiation and non-irradiated but treated groups were included in all experiments.

### Quantitative Real-Time PCR

Total RNA was isolated from undifferentiated NPCs using the ReliaPrep-Kit (Promega, Madison, WI, United States) according to the manufacturer’s protocol. Amount and purity of the isolated RNA was measured with a NanoDrop spectrophotometer ND-100 (Peqlab Biotechnologie GmbH, Erlangen, Germany). One microliter RNA was transcribed into cDNA with qScript cDNA SuperMix (Quantabio, Beverly, MA, United States) according to the manufacturers protocol. Primers were designed using Primer3web and synthesized by Microsynth (Balgach, Switzerland). Efficiency has been performed with serial dilution and an efficiency of 2.0 ± 0.15 was taken as sufficient. Primers are listed in Table 1. For quantification, cDNA was diluted 1:50. All experiments were done in a 7500 real-time PCR cycler (Applied Biosystems, Foster City, CA, United States) with SYBR Green Master Mix (Promega) according to the manufacturer’s protocol. Relative gene-expression was calculated as 2^–ΔΔ*CT*^ values by normalizing to the house-keeping genes GAPDH and β-actin.

**TABLE 1:** Primer sequences used for qRT-PCR.

	**Sequence**
GAPDH fw	5′-TGC ACC ACC AAC TGC TTA-3′
GAPDH rev	5′-GGA TGC AGG ATG ATG TTC-3′
β-actin fw	5′-GAG TGG GGC TTT CGA GTG AT-3′
β-actin rev	5′-AAA GAA AGC CGT GTG CCT TG-3′
S1PR1 fw	5′-ACT ACA CAA CGG GAG CAA CAG-3′
S1PR1 rev	5′-GAT GGA AAG CAG GAG AG-3′
S1PR3 fw	5′-TTC CCG ACT GCT CTA CCA TC-3′
S1PR3 rev	5′-CCA ACA GGC AAT GAA CAC AC-3′
S1PR5 fw	5′-CTT AGG CCT GGA AAC C-3′
S1PR5 rev	5′-CCC GCA CCT GAC AGT AAA TC-3′

### Fluorescence-Activated Cell Sorting Analysis (FACS Analysis)

Undifferentiated and differentiated cells were washed with PBS and centrifuged for 10 min at 1200 rpm. The pellet was incubated with 0.5 μg anti-S1P1 (R&D Systems, Minneapolis, MN, United States) in PBS for 15 min at 4°C. After a washing step, cells were resuspended in PBS and measured with a BD FACSCanto II (Franklin Lakes, NJ, United States) and analyzed by FlowJo software (Treestar, Ashland, OR, United States). For every measurement an unstained control was included.

### Immunocytochemistry

The *in vitro* experiments were terminated 24 or 96 h after irradiation. After fixation with 4% (PFA; Acros, Geel, Belgium), the cells were stained with primary antibodies against Ki67 (1:150, Thermo Fisher Scientific), activated caspase-3 (1:1000, Sigma-Aldrich), DCX (1:250, Santa Cruz Biotechnology, Santa Cruz, CA, United States) and βIII-tubulin (1:1000, Sigma-Aldrich) and the corresponding fluorescent secondary antibody (Alexa Fluor 488 or ALEXA Fluor 555, Life Technologies). For each staining a negative control with only secondary antibody was done. The coverslips were mounted on slides with DAPI-Fluoromount (Biozol, Eching, Germany) and analyzed with a fluorescent microscope (Olympus BX51, Germany). PI (Life Technologies) staining was done on viable cells. Cells were incubated with PI (1:50) and Hoechst 33342 solution (4 μg/ml, Thermo Fisher Scientific) and analyzed afterward with a fluorescent microscope. For quantification, the number of positive cells was counted with the software ImageJ software 1.46r and the percentage of total cell number was calculated and normalized to their non-irradiated counterparts. In every experiment, six fields of view per condition were taken randomly with an 20× objective.

### Cranial Irradiation of Adult Mice

For *in vivo* experiments 9–10 weeks old C57BL/6 wildtype mice were matched into three groups with an equal distribution of age and gender. One group received once daily 1 mg/kg bodyweight FTY720 (Cayman Chemical) solved in water by oral gavage until the end of the experiment. A second group only received water once daily by oral gavage. One week after start of preventive treatment, mice of both groups were exposed to cranial irradiation by an X-ray machine RT250 (CHF Müller) at the RUBION. The mice received a single dose of 6 Gy with 220 kv and 7 mA. The third group of sham-irradiated mice was used as a control for the irradiation process and received neither FTY720 nor H_2_O. For irradiation, mice were anesthetized by an injection of 10% Ketamine and 2% Xylanzin (10 μl/kg bodyweight) and placed into a custom-built lead box with a 1 cm diameter hole over the head to ensure an exclusive cranial irradiation (Supplementary Figure 1). The experiments were terminated 6 h, 24 h, or 4 weeks after irradiation. To analyze proliferation, mice received a single injection of (BrdU, 200 mg/kg, Sigma-Aldrich) 24 h before perfusion. Over the whole period daily body weight was measured and behavior and physical appearance recorded.

### Bioluminescence Imaging

For Bioluminescence imaging (BLI), 8- to 10-week-old B6(Cg)-Tyrc-2J/J DCX-luc reporter mice were injected intraperitoneally with 150 mg/kg D-Luciferin (Synchem, Germany) and anesthetized with 2% isoflurane. Serial images were taken from 5 to 20 min post injection (acquisition time: 59 s, f-stop: 1, binning: 2, field of view: D) with the IVIS Lumina II Imaging System (PerkinElmer, United States). The photon flux (photons/s/cm^2^/steradian) was calculated for the head region and the spinal cord, kept in a constant area and position, using the LIVINGIMAGE software (PerkinElmer, United States). The maximum photon emission was determined from the acquisition of the signal-time curve, recorded with 59 sec temporal resolution, and corrected for background. Prior to each experiment two separate baseline measurements were done and the average calculated. Over a period of 28 days mice received a daily treatment with either 1 mg/kg bodyweight FTY720 (Cayman Chemical) or water as a control.

### Histological Analysis of Neurogenic Niches

At the end of the experiments, mice were transcardially perfused with PBS and 4% PFA, brains were removed and incubated in 4% PFA for 24 h at 4°C. Afterward brains were transferred into 30% sucrose for 48 h and 20 μm sagittal sections of the left hemisphere were made with a microtome (Microm HM 550, Thermo Fisher Scientific). For immunohistochemistry every tenth section was stained against DCX (1:250, Santa Cruz Biotechnology), BrdU (1:1000, Sigma-Aldrich), caspase-3 (1:200, Sigma-Aldrich), Iba1 (1:750, Wako, Tokyo, Japan), GFAP (1:750, Dako, Glostrup, Denmark), and CD3 (1:100, Bio-Rad, Hercules, CA, United States) and their corresponding secondary antibodies Alexa Fluor 488 or Alexa Fluor 555 (1:1000, Life Technologies). For each staining a negative control with only secondary antibody was done. The spleen was taken as positive control for the CD3 staining. All slices were mounted with DAPI-Fluoromount (Biozol).

The expression of DCX and BrdU in the DG and the SVZ was visualized and counted in every tenth slice (14–15 slices per animal) with a fluorescence microscope (Olympus BX51, Germany). The total number of cells was multiplied with 10 and duplicated subsequently to account for the two hemispheres. The amount of Iba1 staining was analyzed by calculating the percentage of positive cells in the entire DG at bregma 1.08 mm. Pictures were made with a fluorescence microscope (Olympus BX51) and cells were counted with ImageJ software 1.46r. For GFAP staining the integrated density was measured in two different areas of the DG. Images were made with a fluorescence microscope of every tenth slice and analyzed with ImageJ software 1.46r.

### Statistical Analysis

Data are shown as mean ± SD or SEM. Two groups were analyzed using Mann–Whitney test. Comparisons between three groups were done with one-way ANOVA with Dunnett’s *post hoc* test when data were normally distributed. Otherwise Kruskal–Wallis test with correction for Dunn’s multiple comparison test was performed. For statistical analysis GraphPad Prism 6.0 (GraphPad Software, San Diego, CA, United States) was used and *p* < 0.05 was considered to be significant.

## Results

### Pretreatment of NPC With FTY720 Reduces Radiation-Induced Cell Death

**FIGURE 1:**
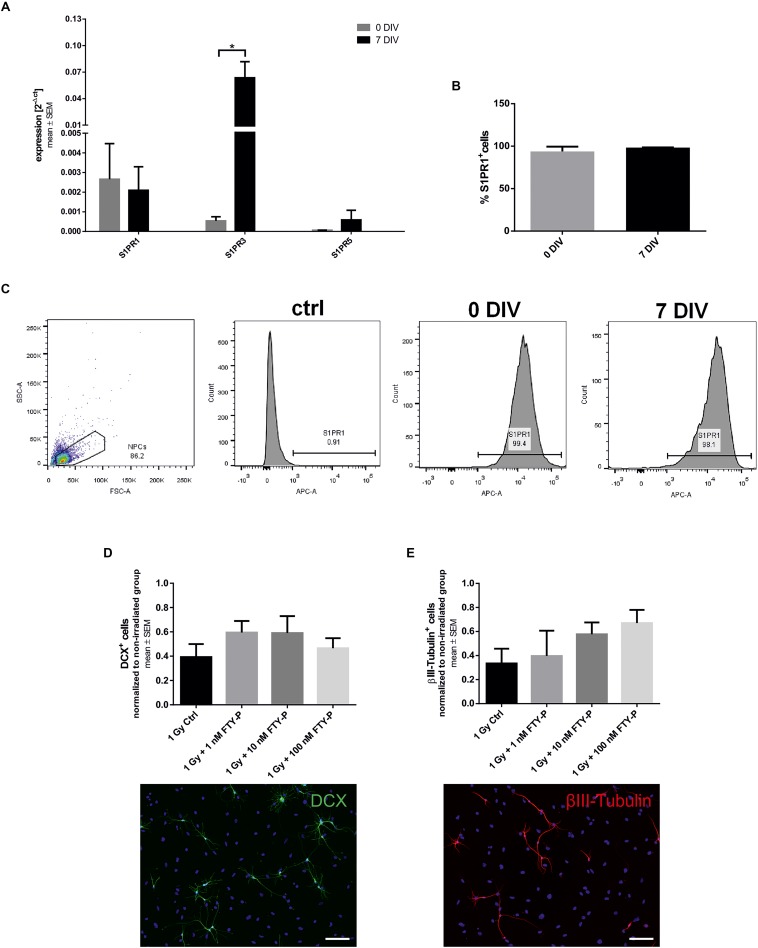
Sphingosine-1-phosphate receptor expression and differentiation of FTY720P-treated neuronal stem cells. **(A)** Embryonic neural precursor cells (NPCs) were explanted from E13 C57BL/6 wildtype mice and cultured as neurospheres. The mean expression level of the sphingosine-1-phosphate receptors S1PR1, S1PR3, and S1PR5 on undifferentiated NPCs (0 days *in vitro*, DIV) and on NPCs after 7 days of differentiation (7 DIV) was analyzed by qt-PCR and normalized to GAPDH and β-actin (mean ± SEM, S1PR1, S1PR5: *n* = 3, S1PR3 *n* = 4). **(B)** Cells were stained against S1PR1 and analyzed via fluorescence-activated cell sorting (FACS). **(C)** Representative pictures of the FACS gating strategy and of the unstained control. **(D,E)** NPCs were treated with different concentrations of FTY720P beginning on day 0 of differentiation and received an irradiation of 1 Gy after 6 days. Differentiation was analyzed 24 h after irradiation by immunocytochemistry with doublecortin (DCX, **B**; 24 h: *n* = 4) and βIII-tubulin (**C**; 24 h: *n* = 3). Irradiated groups were normalized to their sham irradiated counterparts. Results are presented as mean ± SEM. N represents the number of independent experiments. Statistical analysis with Mann–Whitney test **(A)** or Kruskal–Wallis **(B,D,E)**. *p < 0.05. Representative examples of each staining are shown at the bottom, scale = 100 μm.

In a first step, we investigated whether undifferentiated NPCs obtained from E13 mice express the receptors S1PR1, S1PR3, and S1PR5, which are all potential targets of FTY720. All three S1P-receptors were detectable on NPCs (Figure 1A). The highest expression level was shown for S1PR1 (0.002656 ± 0.001813), the expression of S1PR3 was more than threefold lower (0.0005388 ± 0.0002118), and the lowest expression was found for S1PR5 (6.03 × 10^-5^ ± 0.559 × 10^-5^). After 7 days of culture in differentiation medium, there was a significant increase of the S1PR3 (118-fold, *p* = 0.028). Nearly all of the utilized cells were positive for S1PR1 at day 0 (93%) and day 7 after differentiation (98%, Figures 1B,C).

To investigate the impact of FTY720 on differentiation of NPCs we treated them with FTY720P (1, 10, and 100 nM) in differentiation medium. At day 6, the cells received a single dose of 1 Gy X-ray radiation and 24 h later immunocytochemical staining against two different neuronal markers was done. Doublecortin (DCX) is a marker for neuronal precursor cells, whereas βIII-tubulin is expressed by more mature neurons (Ming and Song, 2011). Under control conditions 7.42 ± 3.15% DCX^+^ and 11.65 ± 3.06% βIII-tubulin^+^ cells were found in culture. Twenty four hours after irradiation DCX^+^ (Figure 1D) and βIII-tubulin^+^ (Figure 1E) cells decreased by 60%. Pretreatment with FTY720P did not significantly change the number of DCX^+^ cells or of βIII-tubulin^+^ mature neurons. The expression level of S1PR1 and S1PR3 on NPCs was not altered by irradiation, whereas the expression of S1PR5 decreased (data not shown).

**FIGURE 2:**
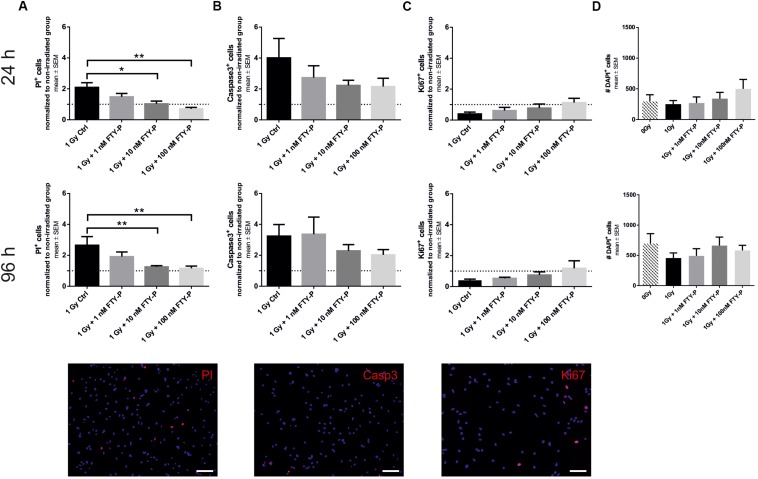
Cell death and proliferation of FTY720P-treated neuronal stem cells. Cell death and proliferation was analyzed 24 and 96 h after irradiation by immunocytochemistry with propidium iodide (PI) (**A**; 24 h: *n* = 6, 96 h: *n* = 6), active caspase-3 (**B**; 24 h: *n* = 5, 96 h: *n* = 6), Ki67 (**C**; 24 h: *n* = 5, 96 h: *n* = 6). **(D)** Total number of DAPI positive cells was calculated for both time points (*n* = 6). Results are presented as mean ± SEM. Irradiated groups were normalized to sham irradiated counterparts. ^*^p < 0.05; ^∗∗^*p* < 0.01 (one-way ANOVA: **B** 96 h, **D** 96 h; Kruskal–Wallis: **A,B** 24 h, **C,D** 24 h). Representative cells examples of each staining are shown at the bottom, scale = 100 μm.

Next, we analyzed the effects of FTY720P on apoptosis and proliferation *in vitro*. 24 h after irradiation with 1 Gy there was a twofold increase in NPCs stained with the dye PI, indicating dead cells, in comparison to sham-irradiated cells (Figure 2A). The percentage of PI+ cells in culture increases from 16.72 ± 3.32 to 33.96 ± 4.29%. Pretreatment with 10 and 100 nM FTY720P led to a significant reduction of PI-positive cells in a dose-dependent manner (*p* = 0.038 and *p* < 0.001, respectively). Also 96 h after irradiation there was an increase of cell death from 10.44 ± 2.76 to 28.06 ± 11.91% by irradiation. In all three treated conditions, an amelioration of cell death could be observed which was significant in the 10 and 100 nM FTY720P group (*p* = 0.004). There was a fourfold increase of activated caspase-3 positive apoptotic cells 24 h after irradiation (2.52 ± 0.83%) in comparison to sham-irradiated NPCs (7.04 ± 1.02%) (Figure 2B). Although there was a dose-dependent reduction of caspase-3 staining with FTY720P pretreatment, no significant changes were found 24 and 96 h after irradiation. In a next step, we analyzed the influence of FTY720P on the proliferation of NPCs using Ki67. Irradiation with 1 Gy led to a 50% reduction of proliferating cells after 24 h from 7.63 ± 2.92 to 4.21 ± 2.09% (Figure 2C). Pretreatment with FTY720P caused an increase of proliferation to levels of non-irradiated control cells, which, however, was not statistically significant. 96 h after irradiation the percentage of Ki^+^ cells was reduced from 11.53 ± 3.32 to 5.08. ± 2.36%. There was no significant difference in the number of DAPI^+^ cells between non-irradiated and irradiated groups (Figure 2D).

### Radiation-Induced Inhibition of Neurogenesis Is Partly Rescued by FTY720

**FIGURE 3:**
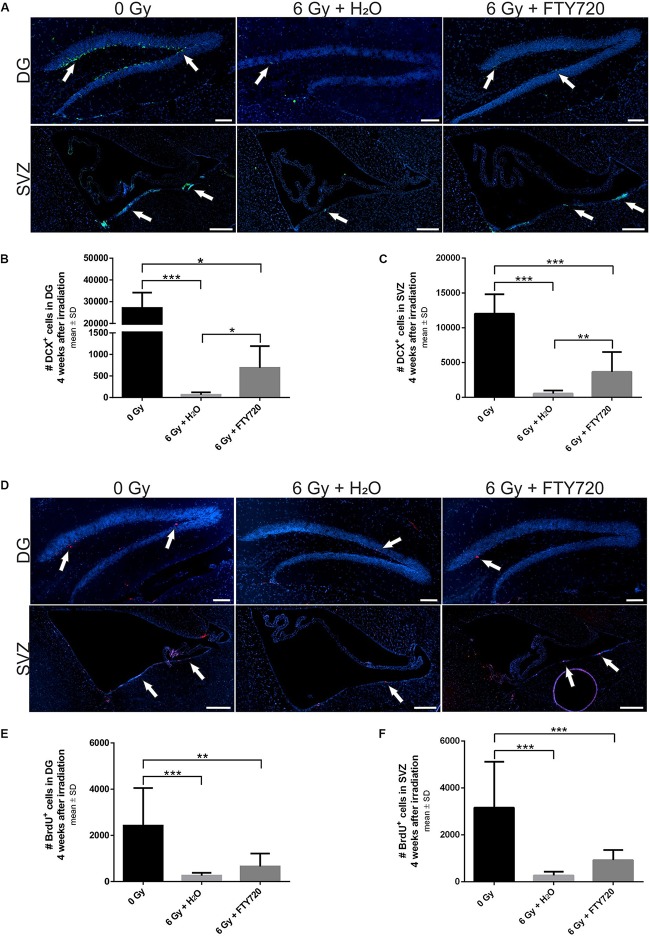
Effect of FTY720 treatment on neurogenesis in adult mice 4 weeks after cranial irradiation. Mice were treated daily with FTY720 (1 mg/kg), received a whole brain irradiation with a single dose of 6 Gy after 7 days, and were sacrificed 4 weeks later. **(A)** Representative images of doublecortin positive (DCX^+^) neurons in the dentate gyrus (DG, top) and the subventricular zone (SVZ, bottom). **(B,C)** Number of DCX^+^ cells in the DG and the SVZ. **(D)** Representative images of proliferating cells in the DG (top) and the SVZ (bottom). Twenty four hours before perfusion all mice received a single dose of bromodeoxyuridine (BrdU, 200 mg/kg). **(E,F)** Number of BrdU^+^ cells in the neurogenic niches DG and SVZ. 0 Gy: *n* = 12 mice, 6 Gy + H_2_O: *n* = 11 mice, 6 Gy + FTY720: *n* = 12 mice. Results are presented as mean ± SD. ^*^p < 0.05; ^∗∗^*p* < 0.01; ^∗∗∗^*p* < 0.001 (one-way ANOVA: **C,F**; Kruskal–Wallis test: **B,E**). Scale = 100 μm (DG) or 200 μM (SVZ). White arrows indicate DCX^+^ **(A)** and BrdU^+^ cells **(D)**.

To examine potential neuroprotective effects of FTY720 *in vivo*, adult C57BL/6 mice received a single dose of 6 Gy whole brain radiation after 1 week of preventive FTY720 treatment. Sham-irradiated as well as H_2_O-treated and irradiated control groups were included. The experiment ended 4 weeks after irradiation. During this period, treated mice received a daily dose of 1 mg/kg FTY720 or H_2_O. For analyzing the effect on neural stem cells in the neurogenic niches of the DG and SVZ, immunostainings for the differentiation marker DCX and the proliferation marker BrdU were done (Figure 3).

Irradiation led to a significant loss of DCX^+^ cells in the DG [*p* < 0.001; *F*(2,32) = 30.28] and in the SVZ [*p* < 0.001; *F*(2,32) = 8.134] 4 weeks after irradiation (Figures 3A–C). Daily treatment with FTY720 ameliorated the loss of DCX^+^ neurons significantly in the DG [*p* = 0.02; *F*(2,32) = 30.28] and in the SVZ [*p* = 0.003; *F*(2,32) = 8.134] (Figures 3B,C). Proliferation was also significantly decreased by irradiation in the DG [*p* < 0.001; *F*(2,32) = 23,26] and in the SVZ [*p* < 0.001; *F*(2,32) = 12.87] after 4 weeks (Figures 3D–F). There was only a slight increase of BrdU^+^ cells with FTY720 pretreatment in the DG [*p* = 0.2766; *F*(2,32) = 23,26] and the SVZ [*p* = 0.3884; *F*(2,32) = 12.87], which failed statistical significance (Figures 3E,F).

**FIGURE 4:**
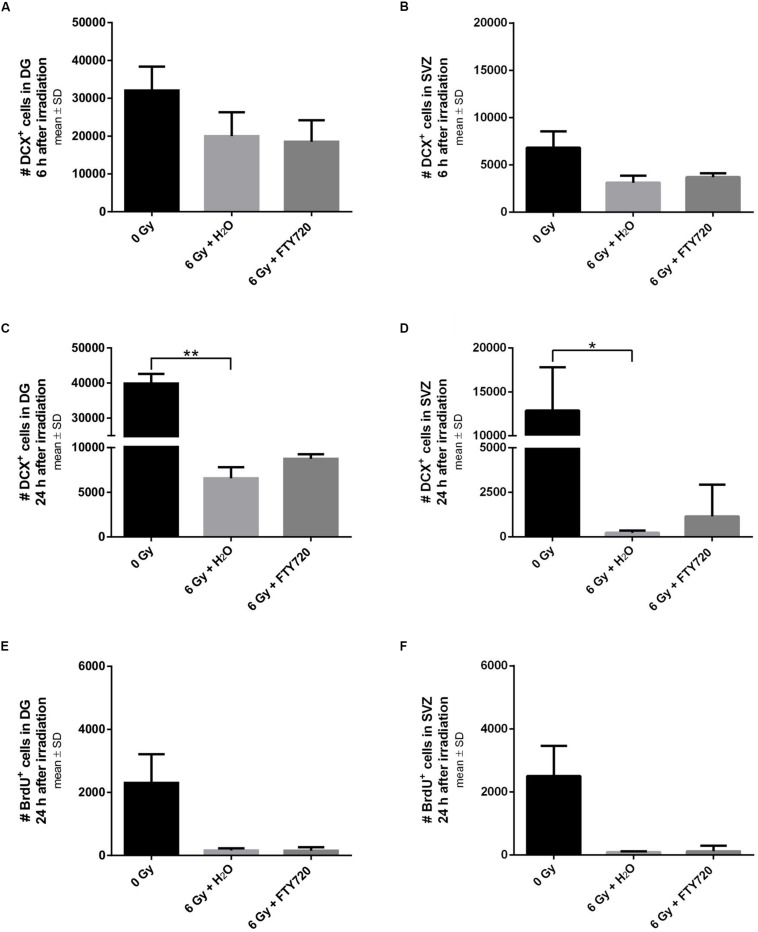
Neurogenesis and proliferation in neurogenic niches 6 and 24 h after irradiation. Mice were treated daily with FTY720 (1 mg/kg), received a whole brain irradiation with a single dose of 6 Gy after 7 days, and were sacrificed 6 or 24 h later. **(A,B)** Quantification of doublecortin positive (DCX^+^) cells in the DG (*n* = 3 mice) and the SVZ (*n* = 3 mice) 6 h after irradiation. **(C,D)** Number of DCX^+^ cells in the DG (0 Gy: *n* = 3 mice, 6 Gy + H_2_O: *n* = 4 mice, 6 Gy + FTY720: *n* = 4 mice) and the SVZ (0 Gy: *n* = 3 mice, 6 Gy + H_2_O: *n* = 4 mice, 6 Gy + FTY720: *n* = 4 mice) 24 h after irradiation. **(E,F)** Number of bromodeoxyuridine positive (BrdU^+^) cells in the neurogenic niches DG and SVZ (0 Gy: *n* = 3 mice, 6 Gy + H_2_O: *n* = 4 mice, 6 Gy + FTY720: *n* = 4 mice). Results are presented as mean ± SD. ^*^p < 0.05; ^∗∗^*p* < 0.01 (Kruskal–Wallis test).

To investigate temporal changes of markers for neurogenesis and proliferation in FTY720 pretreated mice, we also did brain histology 6 and 24 h after irradiation (Figure 4). Loss of DCX^+^ neurons started very fast after irradiation. A decline was observed already 6 h after irradiation in the DG [*p* = 0.2211; *F*(2,6) = 5.6] and in the SVZ [*p* = 0.0513; *F*(2,6) = 5.956] (Figures 4A,B), resulting 24 h after irradiation in a significant decrease in the DG [*p* = 0.0051, *F*(2,8) = 8.909] and in the SVZ [*p* = 0.0462; *F*(2,8) = 6.313] (Figures 4C,D). Neither 6 nor 24 h after irradiation, pretreatment with FTY720 had a significant effect on the number of DCX^+^ neurons. 24 h after irradiation proliferating NPCs were virtually absent in the DG [*p* = 0.8348; *F*(2,8) = 6.158] and in the SVZ [*p* = 0.2019; *F*(2,8) = 6.587], with no change by FTY720 pretreatment (Figures 4E,F).

### Microglia Activation Is Reduced by Pretreatment With FTY720

**FIGURE 5:**
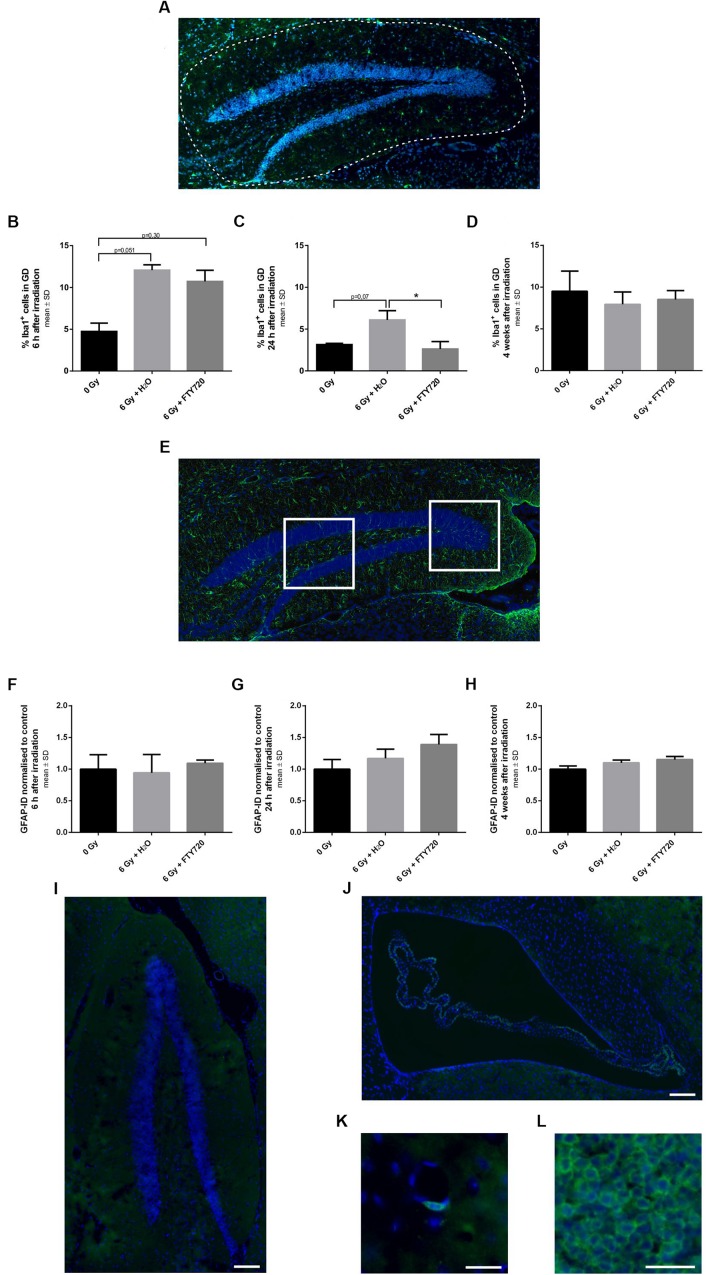
Inflammation in the dentate gyrus of adult mice after 6 Gy irradiation. Mice were treated daily with FTY720 (1 mg/kg), received a whole brain irradiation with a single dose of 6 Gy after 7 days, and were sacrificed 6 h, 24 h, or 4 weeks later. **(A)** Representative picture of a staining against Iba1 a marker for activated microglia. The dotted line indicates the analyzed area. Percentage of Iba1^+^ cells was calculated 6 h (**B**, *n* = 3 mice), 24 h (**C**, 0 Gy: *n* = 3 mice, 6 Gy + H_2_O: *n* = 4 mice, 6 Gy + FTY720: *n* = 4 mice) and 4 weeks after irradiation (**D**, 0 Gy: *n* = 12 mice, 6 Gy + H_2_O: *n* = 11 mice, 6 Gy + FTY720: *n* = 12 mice). **(E)** Representative image of GFAP^+^ cells in the dentate gyrus (DG). The white fields assign the two analyzed areas. Integrated density for each animal was examined 6 h (**F**, *n* = 3 mice), 24 h (**G**, 0 Gy: *n* = 3 mice, 6 Gy + H_2_O: *n* = 4 mice, 6 Gy + FTY720: *n* = 4 mice) and 4 weeks after irradiation (**H**, 0 Gy: *n* = 12 mice, 6 Gy + H_2_O: *n* = 11 mice, 6 Gy + FTY720: *n* = 12 mice). **(I–L)** Immunhistological staining against CD3 shows no infiltration of T cells in the dentate gyrus and the subventricular zone of irradiated animals treated with H_2_O at any time point. Representative images of the DG **(I)** and the SVZ **(J)**. Few positive cells were detected around blood vessels **(K)** in all conditions. **(L)** Spleen tissue for positive control. Scale = 100 μm **(I,J)**, 25 μm **(K)**, or 50 μm **(L)**. Results are presented as mean ± SD. ^*^p < 0.05 (one-way ANOVA: **D,H**; Kruskal–Wallis test: **B,C,F,G**).

To investigate whether the neuroprotective effect of FTY720 after irradiation was associated with its anti-inflammatory mode of action, we stained brain slices against the activation markers Iba1 (microglia), GFAP (astrocytes) and CD3 (lymphocytes) and analyzed the tissue at various time points after irradiation (Figure 5).

Microglia activation peaked at 6 h after irradiation with a reduction after 24 h. While there was no difference in microglia activation in the DG between FTY720 treated and control mice 6 h after irradiation (Figure 5B), 24 h after irradiation the percentage of activated microglia in the DG was significantly decreased in the FTY720 pretreated group as compared to the H_2_O group [*p* = 0.04; *F*(2,8) = 7.423], and was similar to the non-irradiated control group (Figure 5C). Four weeks after irradiation, the percentage of activated microglia was similar in irradiated and non-irradiated mice [*F*(2,32) = 1.636] (Figure 5D). At no time point after irradiation, there was a change of GFAP as marker for astrocyte activation in the DG (Figures 5E–H). We also checked for T cells in the hippocampus and the SVZ because FTY720 is known to prevent T cell egress from the lymph nodes to the CNS. Staining against CD3 showed no positive cells in the DG or SVZ in irradiated FTY720- or H_2_O-treated or of non-irradiated mice at any time point (Figures 5I,J). Only a few cells were found around blood vessels in all conditions (Figure 5K). The spleen was used as a positive control (Figure 5L).

### No Increase of Neurogenesis by FTY720 Treatment in Naïve Mice

**FIGURE 6:**
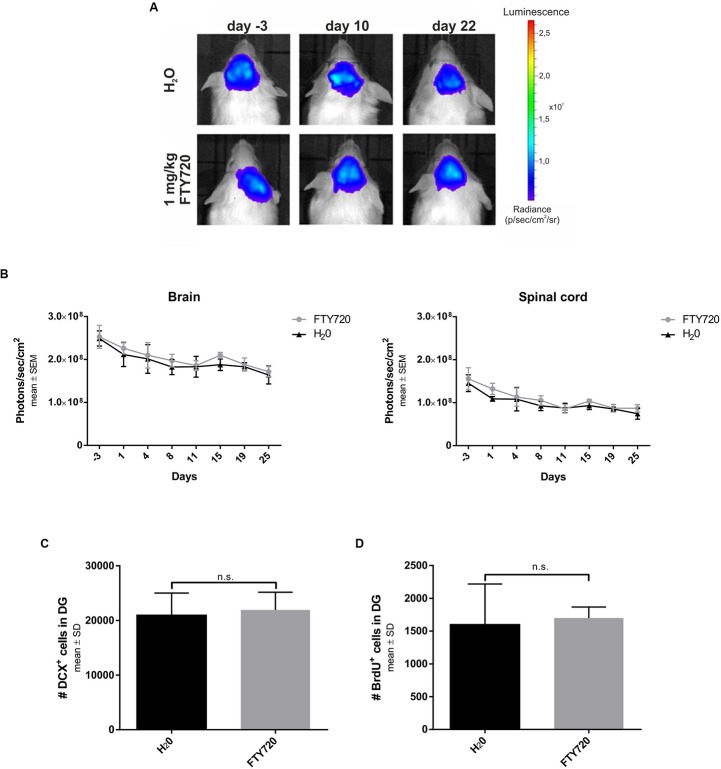
Longitudinal evaluation of neurogenesis in naive mice treated with FTY720. Naive B6(Cg)-Tyrc-2J/J DCX-luc reporter mice were treated from day 0 to 28 with FTY720 1.0 mg/kg (*n* = 5) or water (*n* = 5) by oral gavage once per day. Treatment groups were sex- and age-matched and stratified according to their bioluminescence signal intensity at day −3. **(A,B)** Doublecortin (DCX) signal intensity was evaluated by bioluminescence imaging of areas of interest (brain, spinal cord) twice per week. **(C,D)** After 28 days mice were perfused with PFA and consecutive sagittal brain slices examined by immunohistochemistry for DCX and bromodeoxyuridine (BrdU) in the dentate gyrus. Results are presented as mean ± SEM **(B)** and mean ± SD **(C,D)**.

Finally, we evaluated the possibility that FTY720 treatment *per se* induces neurogenesis. To this end, we treated naive B6(Cg)-Tyrc-2J/J DCX-luc reporter mice, which can be used to visualize neurogenesis longitudinally (Ayzenberg et al., 2015), with 1 mg/kg FTY720 for 4 weeks in the absence of irradiation (Figure 6). Bioluminescence imaging revealed no differences in neurogenesis in the brain and the spinal cord between treated and untreated mice throughout the observation period (Figures 6A,B). In line with this finding, the number of DCX^+^ cells (H_2_O: 21920 ± 3248; FTY720: 20948 ± 4056) and of BrdU^+^ proliferating cells in the DG (H_2_O: 1700 ± 373; FTY720: 1600 ± 619) was unchanged after 4 weeks of treatment (Figures 6C,D).

## Discussion

In this study, we investigated whether a preventive treatment with FTY720 protects NPCs from irradiation-induced damage. We show, that FTY720 treatment of NPCs prior to X-ray exposure leads to an ameliorated cell death *in vitro* and *in vivo* and therefore is neuroprotective. Furthermore, we could demonstrate an inhibition of microglia activation in the DG 24 h after X-ray exposure. No effects on neurogenesis were found.

Sphingosine-1-phosphate receptors are expressed on many cells of the CNS and play an important role in neuronal development and angiogenesis. Mizugishi et al. (2005) observed embryonic lethality in S1P deficient mice due to decreased mitosis and increased cell death. They also demonstrated the involvement of S1PR1 in this process (Mizugishi et al., 2005). S1P induces proliferation of cultured hippocampal precursors by induction of MAP kinase pathways (Harada et al., 2004) and enhances NPC migration to the area of injury in an infarcted brain (Kimura et al., 2008). We analyzed the NPC-restricted expression pattern of the receptors S1PR1, S1PR3, and S1PR5, which are the main expressed receptors in the CNS (Groves et al., 2013). We demonstrate that NPCs express all three receptors on a different level and therefore are potential targets of FTY720.

FTY720 is known to have neuroprotective effects (Ayzenberg et al., 2016), but its effects on NPCs were not well characterized. We demonstrate that irradiation of differentiated NPC cultures with 1 Gy X-rays resulted in a rapid increase of cell death and reduced proliferation. Treatment with 100 nM FTY720 led to a significant reduction of cell death 24 and 96 h after irradiation *in vitro*. Our results are in line with previous studies, which have shown a protective potential of FTY720 to X-ray exposure using *in vitro* assays. Stessin et al. (2012) observed an increased cell viability and differentiation of adult stem cells upon FTY720 treatment after irradiation.

Since there was no significant change in the amount of apoptotic cells in our experiments, we conclude that the neuroprotective effect of FTY720 pertains to other types of cell death, particularly necrosis or autophagy. Similarly, in a model of traumatic brain injury, Zhang et al. (2016) observed an increased expression of the autophagy markers LC3 and Beclin1 in mice, which could be partly abolished by the inhibitor 3-methyladenine. In contrast to Stessin et al., who showed an ameliorated neuronal differentiation 72 h after 6 Gy radiation, we could not detect an enhanced neuronal differentiation in FTY720-treated NPCs. Sun et al. (2016) also reported no influence on stem cell differentiation *in vitro*, but they observed enhanced proliferation of NPCs treated with 100 and 250 nM FTY720. In our study we could show only a tendency of increased proliferation in the 100 nM group. Perhaps higher doses of FTY720, for example, 200 or 500 nM, would enhance this effect.

Importantly, our *in vivo* results, showing an increased number of neuronal progenitor cells in neurogenic niches of the brain 4 weeks after 6 Gy X-ray exposure, indicate a neuroprotective effect of FTY720. These results are in line with a recent study, which demonstrated partial restauration of neurogenesis in the DG 7 weeks post-irradiation (Stessin et al., 2017). Since FTY720 pretreatment did not have significant effects on the DCX^+^ cells and on proliferation in the first 24 h after irradiation, we conclude that neuroprotection by FTY720 mitigates delayed damage. In contrast to other studies using higher doses in healthy mice (Sun et al., 2016), daily treatment of FTY720 (1 mg/kg) did not induce neurogenesis, favoring a neuroprotective mode of action toward irradiation-induced damage. Perhaps higher doses will enhance this neuroprotective effect, which could be analyzed in future. As a limitation of our study, the number of mice used in the *in vivo* experiments was limited and should be increased in prospective studies to enhance statistical power in these experiments.

Other studies have shown neuroprotective effects of FTY720 *in vivo*. Using a model of excitotoxic cell death, Di Menna et al. (2013) observed an enhanced survival of cortical neurons treated with FTY720 prior to the administration of NMDA. This effect was mediated by MAP-kinase and PtdIns-3-kinase pathways through activation of the S1P1 receptor (Di Menna et al., 2013). Furthermore, the intracerebro-ventricular injection of kainic acid and of FTY720, followed by a daily treatment of 1 mg/kg FTY720 i.p., resulted in an increased number of DCX/BrdU^+^ cells in the DG of adult rats in another study (Cipriani et al., 2017).

In experimental models of neurodegenerative diseases, potential neuroprotective effects of FTY720 were also studied. In the MPTP-model, a model of Parkinson’s disease, FTY720 ameliorated the loss of TH^+^ cells through the activation of the Akt-kinase pathway. The authors hypothesized a protection of mitochondria by increased BAD-protein phosphorylation through FTY720, which led to minor activation of apoptotic signaling pathways (Motyl et al., 2018). In a mouse model of Alzheimer’s disease, the treatment with FTY720 reduced amyloid-β-pathology as well as astrocyte and microglia activation (Aytan et al., 2016). In contrast to previous studies (Efstathopoulos et al., 2015; Sun et al., 2016) we could not detect changes of neurogenesis in naïve mice, which might be due to different dosages and times of FTY720 treatment.

Another driver of irradiation-induced brain damage could be inflammation, especially microglia and macrophages (Lumniczky et al., 2017). Studies have shown that whole brain irradiation leads to microglial activation, which correlates with neuronal cell death (Monje et al., 2002, 2003). *In vitro* irradiation of a murine microglia cell line increased microglia activation resulting in a release of inflammatory cytokines like interleukin 6 (IL-6) and tumor necrosis factor α (TNFα) (Liu et al., 2010). We observed a decreased amount of activated microglia in and around the DG 24 h after irradiation in the FTY720-treated group in comparison to the untreated group. In line with these results, FTY720 treatment in a model of KA-induced injury of the hippocampus led to anti-inflammatory effects on microglia and less neuronal loss (Cipriani et al., 2015). Furthermore, Noda et al. (2013) showed a reduction of the proinflammatory cytokines IL-1β, IL-6, and TNFα by FTY720 treatment after LPS-stimulation of primary microglial cells. They could also observe a significant release of brain-derived neurotrophic factor (BDNF) and of glial cell-derived neurotrophic factor (GDNF) (Noda et al., 2013).

Similar to previous studies (Ben Abdallah et al., 2007), our experiments did not indicate an involvement of astrocytes in the neuroprotective effect of FTY720. In contrast, Colombo et al. (2014) observed a reduction of NFkB activation and of NO synthesis in astrocytes, leading to ameliorated neurodegeneration in the multiple sclerosis model EAE. Other studies investigating cranial irradiation showed an increased astrogliosis when using a high dose (≥10 Gy) exposure (Chiang et al., 1993; Hong et al., 1995; Hwang et al., 2006; Liu et al., 2010).

Finally, we examined whether involvement of immune cell infiltration, particularly of lymphocytes, is involved in the impaired neurogenesis during irradiation. Moravan et al. (2011) observed an increased number of CD3^+^ T cells 30 days after cranial irradiation with 35 Gy. In our study there were no CD3^+^ cells found in the hippocampus or the SVZ. Therefore it is unlikely, that the protective effect is due to the ability of FTY720 to prevent infiltration of lymphocytes into the CNS.

The mechanisms of neuroprotection by FTY720 remains unknown. We could show the expression of S1P1R, S1P3R, and S1P5R on NPCs, but which receptor or downstream pathways are involved have to be analyzed in further studies. A reduction of oxidative stress is a potential mechanistic explanation for the treatment effect of FTY720. Irradiation causes increased oxidative stress leading to suppression of hippocampal neurogenesis (Huang et al., 2012). In a model of H_2_O_2_-induced demyelination, FTY720 attenuated neuronal damage without altering the number of proinflammatory cytokines (O’Sullivan et al., 2017). Additionally, the influence on BDNF levels should be investigated. Cranial irradiation leads to a decrease of BDNF in the hippocampus (Ji et al., 2014; Son et al., 2015a) and studies in murine models of Rett syndrome or depression already have shown that FTY720 treatment increases hippocampal BDNF levels (Deogracias et al., 2012; di Nuzzo et al., 2015).

## Conclusion

Taken together, our study demonstrates protective effects of FTY720 on neuronal stem cells in models of irradiation-induced cell death. Given the devastating effects of whole-brain irradiation, therapies to prevent neuronal damage are an unmet medical need. The approved drug fingolimod might be a therapeutic option to prevent irradiation-induced neurocognitive dysfunction. Its neuroprotective properties might also be of interest for other diseases with a neurodegenerative component, including ischemic stroke, multiple sclerosis or CNS injuries.

## Data Availability

The datasets generated for this study are available on request to the corresponding author.

## Author Contributions

JM designed and performed the experiments, interpreted the data, and wrote the manuscript. ZH, SiS, and IA performed the experiments and interpreted the data. SaS, XP, and SH performed the experiments and participated in the design of the study. RG participated in the design of the study and interpreted the data. LT and IK designed the experiments, interpreted the data, coordinated the study, and wrote the manuscript. All authors read and approved the final manuscript.

## Conflict of Interest Statement

IA received board honoraria from Merck Serono, Roche, travel grant from Biogen Idec and grant support from Chugai Pharma. RG received speaker’s and board honoraria from Baxter, Bayer Schering, Biogen Idec, CLB Behring, Genzyme, Merck Serono, Novartis, Stendhal, Talecris, and TEVA. His department received grant support from Bayer Schering, Biogen Idec, Genzyme, Merck Serono, Novartis, and TEVA. LT received travel funding and/or speaker honoraria from AbbVie, Bayer, Bial, Desitin, GE, UCB, Zambon, and consulted for AbbVie, Bayer, Bial, Desitin, UCB, Zambon. IK received travel funding and/or speaker honoraria from Biogen, Merck, Novartis, Sanofi, Roche, consulted for Bayer Healthcare, Chugai, Roche, Shire, received research support from Chugai, Diamed and is Associate Editor of BMC Neurology. The remaining authors declare that the research was conducted in the absence of any commercial or financial relationships that could be construed as a potential conflict of interest.

## Abbreviations

BrdU: bromodeoxyuridine
CNS: central nervous system
DG: dentate gyrus
DIV: days *in vitro*
EAE: experimental autoimmune encephalomyelitis
EGF: epidermal growth factor
FBS: fetal bovine serum
FGF: fetal growth factor
GFAP: glial fibrillary acidic protein
Iba1: ionized calcium binding adaptor molecule 1
IL: interleukin
KA: kainic acid
LPS: lipopolysaccharide
MPTP: 1-methyl-4-phenyl-l,2,3,6-tetrahydropyridine
NMDA: *N*-Methyl -D-aspartate
NO: nitric oxide
NPC: neural precursor cell
PFA: paraformaldehyde
PI: propidium iodide
TH: tyrosinhydroxylase
TNF: tumor necrosis factor
S1PR: sphingosine-1-phosphate receptor
SVZ: subventricular zone.
