# Supplementary figures and images for: Fingolimod for Irradiation-Induced Neurodegeneration
Source: Front Neurosci. 2019 Jul 9;13:699. doi: 10.3389/fnins.2019.00699 (PMC6633210; doi:10.3389/fnins.2019.00699)

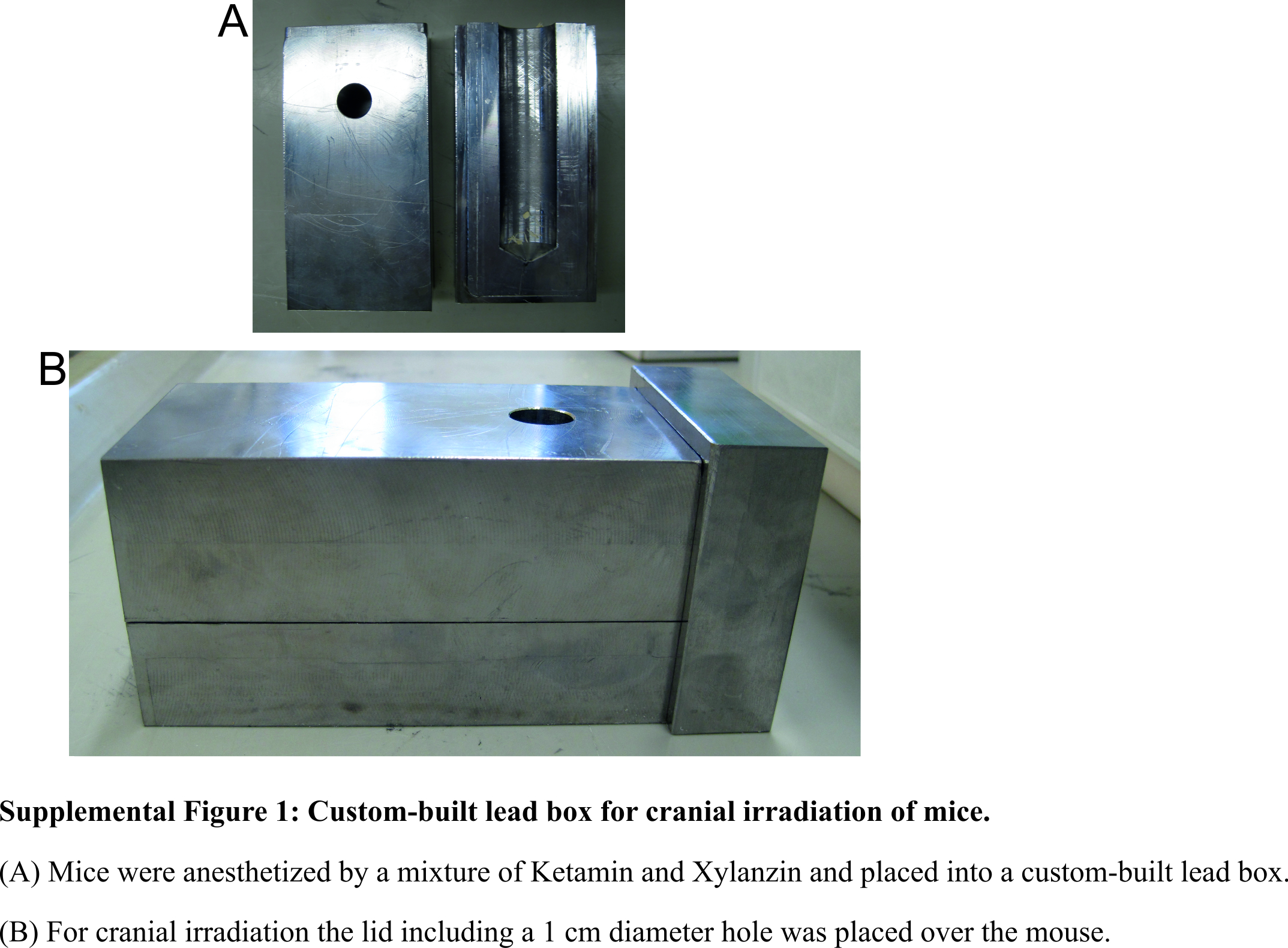

Supplement: Supplementary file 1 [file Image_1.JPEG]
